# Noninvasive Brain Stimulations for Unilateral Spatial Neglect after Stroke: A Systematic Review and Meta-Analysis of Randomized and Nonrandomized Controlled Trials

**DOI:** 10.1155/2018/1638763

**Published:** 2018-06-28

**Authors:** Flávio Taira Kashiwagi, Regina El Dib, Huda Gomaa, Nermeen Gawish, Erica Aranha Suzumura, Taís Regina da Silva, Fernanda Cristina Winckler, Juli Thomaz de Souza, Adriana Bastos Conforto, Gustavo José Luvizutto, Rodrigo Bazan

**Affiliations:** ^1^Neurology Department, Botucatu Medical School, Universidade Estadual Paulista (UNESP), Botucatu, SP, Brazil; ^2^Science and Technology Institute, Universidade Estadual Paulista (UNESP), São José dos Campos, SP, Brazil; ^3^Department of Pharmacy, Tanta Chest Hospital, Tanta, Egypt; ^4^Research Institute, Hospital do Coração (HCor), São Paulo, SP, Brazil; ^5^Neurostimulation Laboratory, University of São Paulo (USP), São Paulo, SP, Brazil; ^6^Department of Applied Physical Therapy, Federal University of Triângulo Mineiro (UFTM), Uberaba, MG, Brazil

## Abstract

**Background:**

Unilateral spatial neglect (USN) is the most frequent perceptual disorder after stroke. Noninvasive brain stimulation (NIBS) is a tool that has been used in the rehabilitation process to modify cortical excitability and improve perception and functional capacity.

**Objective:**

To assess the impact of NIBS on USN after stroke.

**Methods:**

An extensive search was conducted up to July 2016. Studies were selected if they were controlled and noncontrolled trials examining transcranial direct current stimulation (tDCS), repetitive transcranial magnetic stimulation (rTMS), and theta burst stimulation (TBS) in USN after stroke, with outcomes measured by standardized USN and functional tests.

**Results:**

Twelve RCTs (273 participants) and 4 non-RCTs (94 participants) proved eligible. We observed a benefit in overall USN measured by the line bisection test with NIBS in comparison to sham (SMD −2.35, 95% CI −3.72, −0.98; *p* = 0.0001); the rTMS yielded results that were consistent with the overall meta-analysis (SMD −2.82, 95% CI −3.66, −1.98; *p* = 0.09). The rTMS compared with sham also suggested a benefit in overall USN measured by Motor-Free Visual Perception Test at both 1 Hz (SMD 1.46, 95% CI 0.73, 2.20; *p* < 0.0001) and 10 Hz (SMD 1.19, 95% CI 0.48, 1.89; *p* = 0.54). There was also a benefit in overall USN measured by Albert's test and the line crossing test with 1 Hz rTMS compared to sham (SMD 2.04, 95% CI 1.14, 2.95; *p* < 0.0001).

**Conclusions:**

The results suggest a benefit of NIBS on overall USN, and we conclude that rTMS is more efficacious compared to sham for USN after stroke.

## 1. Background

Stroke is the second leading cause of death worldwide and the primary cause of chronic disability in adults [1]. In the United States, it is the fourth leading cause of death overall [2]. Among people who survive a stroke, unilateral spatial neglect (USN) is the most frequent disorder for right hemisphere lesions [3].

The incidence of USN varies widely from 10% to 82% [4, 5]. USN is characterized by the inability to report or respond to people or objects presented on the side contralateral to the lesioned side of the brain and has been associated with poor functional outcomes and long stays in hospitals and rehabilitation centers [6].

Pharmacological interventions such as dopamine and noradrenergic agonists or procholinergic treatment have been used in people affected by USN after stroke, but the evidence derived from a Cochrane systematic review that included only two available RCTs was very low and inconclusive [7].

Other nonpharmacological rehabilitation techniques have been explored for USN with the aim to facilitate the recovery of perception and behavior, which include right half-field eye-patching [8], mirror therapy [9], prism adaptation [10], left-hand somatosensory stimulation with visual scanning training [11], contralateral transcutaneous electrical nerve stimulation and optokinetic stimulation [12], trunk rotation [13], repetitive transcranial magnetic stimulation [14], galvanic vestibular stimulation [15], and dressing practice [16]. However, their results do not support the use of these techniques in isolation for improvement of secondary outcomes such as performance and sensorimotor functions, activities of daily living (ADLs), or quality of life [9, 14, 17].

Noninvasive brain stimulations (transcranial direct current stimulation (tDCS) and repetitive transcranial magnetic stimulation (rTMS)) have already shown their ability to modify cortical excitability [18]. tDCS is a noninvasive method used to modulate cortical excitability by applying a direct current to the brain that is less expensive than repetitive transcranial magnetic stimulation (rTMS). The latter is an electric current that creates magnetic fields that penetrate the brain and can modulate cortical excitability by decreasing or increasing it and potentially improve perceptual and cognitive abilities [19, 20].

A previous Cochrane systematic review summarized results about the effects of tDCS versus control (sham/any other intervention) on activities of daily living (ADLs) among stroke survivors. The authors included 32 randomized controlled trials (RCTs) and concluded that tDCS might enhance ADLs, but upper and lower limb function, muscle strength, and cognitive abilities should be further explored [21]. Another Cochrane systematic review assessed the efficacy of repetitive transcranial magnetic stimulation (rTMS) compared to sham therapy or no therapy for improving function in people with stroke. The 19 included trials showed that rTMS was not associated with a significant increase in ADLs or in motor function; therefore, the authors do not support the use of rTMS for the treatment of stroke, and they plan to complete further trials to confirm their findings [22].

Previous reviews were, however, limited in that they did not include non-RCT studies nor did they evaluate the newest noninvasive brain stimulation—theta burst. We therefore conducted a systematic review of RCT and non-RCT studies that assessed the impact of tDCS, rTMS, and TBS for unilateral spatial neglect after stroke.

## 2. Methods

We adhered to methods described in the Cochrane Handbook for Intervention Reviews [23]. Our reporting also adheres to the Preferred Reporting Items for Systematic Reviews and Meta-Analyses (PRISMA) [24] and Meta-Analysis of Observational Studies in Epidemiology (MOOSE) statements [25].

### 2.1. Eligibility Criteria

The eligibility criteria are as follows:

1. Study designs: RCTs, quasi-RCTs, and non-RCTs
2. Participants: adults over 18 years of age, regardless of gender and the duration of illness or severity of the initial impairment, with USN after any type of stroke diagnosis (ischemic or intracranial hemorrhage) measured by clinical examination or radiographically by computed tomography (CT) or magnetic resonance imaging (MRI), regardless of whether they were included after evaluation by standardized USN tests.
3. Interventions: any noninvasive brain stimulations such as tDCS, rTMS, and including theta burst (continuous TBS (cTBS) or intermittent theta burst (iTBS)) (we considered evaluating both the different types of stimulations (i.e., cathodal tDCS versus anodal tDCS versus dual tDCS) and types of frequency (i.e., high-frequency versus low frequency))
4. Comparators: interventions were to be compared against sham stimulation or any conventional stroke rehabilitation (e.g., pharmacological therapy or nonpharmacological therapy such as right half-field eye-patching, mirror therapy, prism adaptation, left-hand somatosensory stimulation, and visual scanning training or other conventional treatment)

We also considered noninvasive brain stimulations as an adjunct to any type of conventional stroke rehabilitation.

(5) Outcomes:
  (i) Overall USN measured by any paper-and-pencil tests, such as the line cancellation task [26], the line bisection test [27], or the star cancellation test [28], and by any validated specific instrument, such as the Catherine Bergego Scale [29], and the Behavioral Inattention Test [30]
  (ii) Disability in neurological and functional abilities as measured by any validated specific instrument, such as the National Institutes of Health Stroke Scale and the Modified Rankin Scale [31], the box and block test [32], or the Fugl-Meyer Assessment [33] after treatment and over the long term
  (iii) Daily life functions as measured by any validated measurement scale, such as the Barthel index [31]
  (iv) Number of reported falls as measured by diaries of falls, by the Morse Fall Scale [34], or by the Hendrich II Fall Risk Model [35] after treatment and over the long term
  (v) Balance as measured by the Berg Balance Scale, the balance subscale of the Fugl-Meyer test, and the Postural Assessment Scale for Stroke Patients [36] after treatment and over the long term
  (vi) Depression or anxiety as measured by the Beck Depression Inventory, the Hospital Anxiety and Depression Scale, Symptom Checklist-90 (SCL-90), and the Hamilton Depression Rating Scale [37] after treatment and over the long term
  (vii) Evaluation of poststroke fatigue by the Fatigue Severity Scale [38] after treatment and over the long term
  (viii) Quality of life (however defined by the study authors) after treatment and over the long term
  (ix) Adverse events (e.g., euphoria, hallucinations, orthostatic hypotension, nausea, insomnia, dizziness, and syncope) after treatment and over the long term
  (x) Death

### 2.2. Data Source and Searches

We searched MEDLINE (OvidSP) (1966 to July 2016), EMBASE (OvidSP) (1980 to July 2017), the Cochrane Central Register of Controlled Trials (CENTRAL) (The Cochrane Library, 2017, issue 7), CINAHL (1961 to July 2017), and Latin-American and Caribbean Center on Health Sciences Information (LILACS) (from 1982 to July 2017) without language restrictions. The date of the most recent search was 26 July 2017. All searches were conducted with the assistance of a trained medical librarian. We also searched the reference lists of relevant articles and conference proceedings and contacted the authors of included trials.

The search strategy was: (tDCS OR TDCS OR Cathodal Stimulation Transcranial Direct Current Stimulation OR Cathodal Stimulation tDCSs OR Cathodal Stimulation tDCS OR Transcranial Random Noise Stimulation OR Transcranial Alternating Current Stimulation OR Transcranial Electrical Stimulation OR dual transcranial direct current stimulation OR Transcranial Electrical Stimulations OR Anodal Stimulation Transcranial Direct Current Stimulation OR Anodal Stimulation Tdcs OR Anodal Tdcs OR Anodal Stimulation TDCSs OR Repetitive Transcranial Electrical Stimulation OR repetitive transcranial magnetic stimulation OR RTMS OR rTMS OR High-frequency rTMS OR Trasncranial Magnetic Stimulation OR Transcranial Magnetic Stimulations OR Low-frequency transcranial magnetic stimulation OR Stimulation Transcranial Magnetic OR Stimulations Transcranial Magnetic OR Single Pulse Transcranial Magnetic Stimulation OR Paired Pulse Transcranial Magnetic Stimulation OR Repetitive Transcranial Magnetic Stimulation OR theta burst OR theta burst stimulation OR theta-burst OR theta-burst stimulation OR burst stimulation OR continuous theta burst stimulation OR continuous TBS OR TBS) AND (cerebrovascular disorders OR basal ganglia cerebrovascular disease OR hemispatial neglect OR hemispatial neglect OR spatial attentional asymmetries OR brain ischemia OR carotid artery diseases OR intracranial arterial diseases OR intracranial embolism and thrombosis OR intracranial hemorrhages OR stroke OR brain infarction OR vertebral artery dissection OR post-stroke OR poststroke OR hemineglect OR hemi-neglect OR unilateral visuospatial neglect OR visuospatial neglect OR visual spatial neglect OR spatial neglect OR unilateral neglect of acute stroke patients OR unilateral spatial neglect OR patients with stroke OR stroke patients with spatial neglect OR right hemisphere strokes OR rehabilitation after stroke OR chronic spatial neglect after stroke OR unilateral neglect OR spatial neglect OR hemispatial neglect OR visual neglect OR inattention OR hemi-inattention OR space perception OR visual perception OR perceptual disorders OR perceptual disorder OR extinction OR functional laterality).

### 2.3. Selection of Studies

Two pairs of reviewers independently screened all titles and abstracts identified by the literature search, obtained full-text articles of all potentially eligible studies, and evaluated them for eligibility. Reviewers resolved disagreement by discussion or, if necessary, with third party adjudication. We also considered studies reported only as conference abstracts.

### 2.4. Data Extraction

Reviewers underwent calibration exercises and worked in pairs to independently extract data from included studies. They resolved disagreement by discussion or, if necessary, with third party adjudication. They abstracted the following data using a pretested data extraction form: study design, participants, interventions, comparators, outcome assessed, and relevant statistical data.

### 2.5. Risk of Bias Assessment

Reviewers, working in pairs, independently assessed the risk of bias of included RCTs using a modified version of the Cochrane Collaboration's instrument (http:/distillercer.com/resources/) [39]. That version includes nine domains: adequacy of sequence generation, allocation sequence concealment, blinding of participants and caregivers, blinding of data collectors, blinding for outcome assessment, blinding of data analysts, incomplete outcome data, selective outcome reporting, and the presence of other potential sources of bias not accounted for in the previously cited domains [40]. For incomplete outcome data in individual studies, we stipulated as low risk of bias for loss to follow-up as less than 10% and a difference of less than 5% in missing data between intervention/exposure and control groups.

When information regarding risk of bias or other aspects of methods or results was unavailable, we attempted to contact study authors for additional information.

### 2.6. Certainty of Evidence

We summarized the evidence and assessed its certainty separately for bodies of evidence from RCT and non-RCT studies. We used the Grading of Recommendations Assessment, Development and Evaluation (GRADE) methodology to rate certainty of the evidence for each outcome as high, moderate, low, or very low [41]. In the GRADE approach, RCTs begin as high certainty and non-RCT studies begin as moderate certainty. Detailed GRADE guidance was used to assess overall risk of bias [42], imprecision [43], inconsistency [44], indirectness [45], and publication bias [46] and to summarize the results in an evidence profile (Table 1).

We planned to assess publication bias through visual inspection of funnel plots for each outcome in which we identified 10 or more eligible studies; however, we were not able to do so because there were an insufficient number of studies to allow for this assessment.

### 2.7. Data Synthesis and Statistical Analysis

We calculated pooled inverse variance standardized mean difference (SMD) and associated 95% CIs using random-effects models. We addressed variability in results across studies by using *I* ^2^ statistic and the *P* value obtained from the Cochran chi square test. Our primary analyses were based on eligible patients who had reported outcomes for each study (complete case analysis). We used Review Manager (RevMan) (version 5.3; Nordic Cochrane Centre, Cochrane) for all analyses [47].

### 2.8. Subgroup and Sensitivity Analyses

We planned possible subgroup analyses according to the following characteristics:

- Participants (stroke type: ischemic stroke versus intracranial hemorrhage)
- Interventions (type of stimulation: cathodal versus anodal and position of electrodes; type of frequency: high frequency versus low frequency)
- Comparator (type of control intervention: pharmacological therapy versus nonpharmacological therapy)
- Different tests for overall USN (star cancellation test versus line bisection test)

We planned to conduct subgroup analyses only when five or more studies were available, with at least two in each subgroup. We planned to synthesize the evidence separately for bodies of evidence from RCT and non-RCT studies by a sensitivity analysis.

## 3. Results

### 3.1. Study Selection

**Figure 1:**
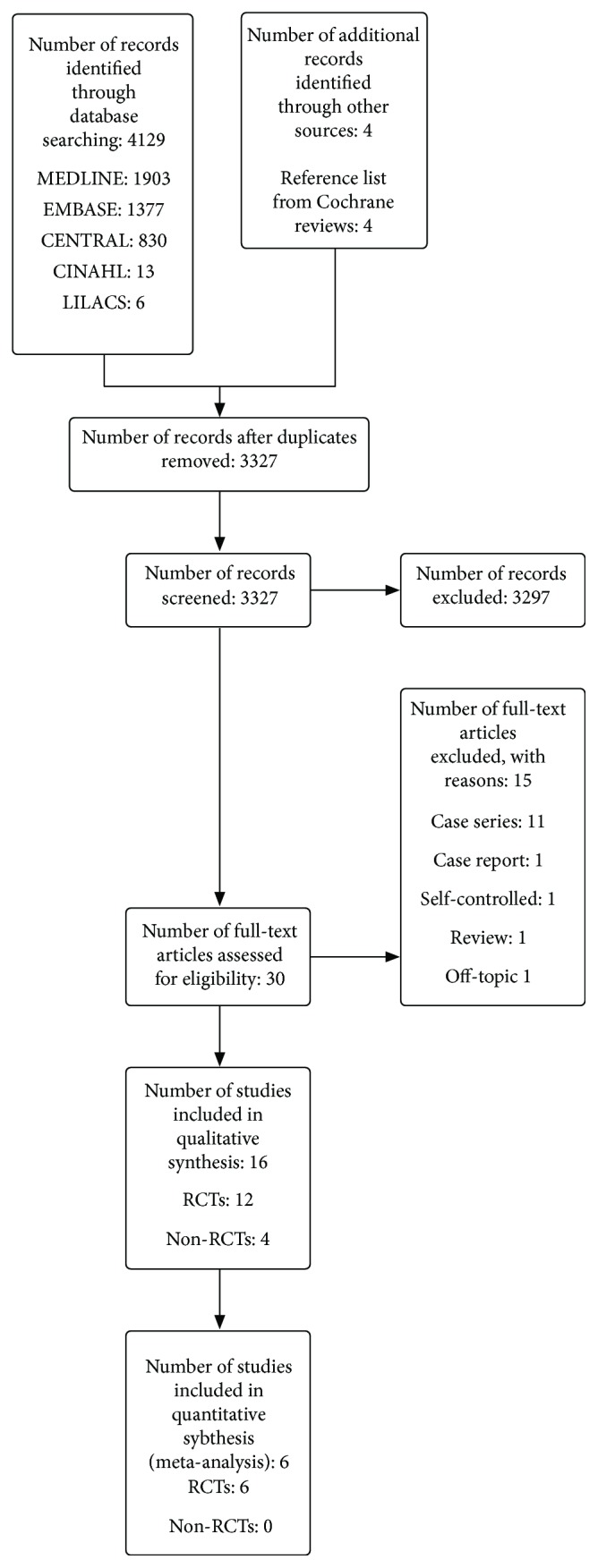
Flow diagram of the systematic review.

We identified a total of 4129 citations through database searches and a further four studies from the reference lists of the Cochrane reviews [22, 48, 49] (see Figure 1 for search results). After screening by title and then by abstract, we obtained full-paper copies for 30 citations that were potentially eligible for inclusion in the review. We excluded 15 studies for the following reasons: case report, case series, self-controlled study, review, and off-topic. The remaining 12 RCTs [14, 50–60] with a total of 273 participants and four non-RCTs [61–64] with a total of 94 participants met the minimum requirements, and we included them in this review.

### 3.2. Study Characteristics

Table 2 describes study characteristics related to design of study, setting, number of participants, mean age, gender, inclusion and exclusion criteria, and follow-up. Eight studies [14, 54, 56, 59, 61–64] were conducted largely in Europe and eight in Asia [50–53, 55, 57, 58, 60]. Randomized trials' sample sizes ranged from 10 [56] to 38 [55], and non-RCT studies ranged from 12 [63] to 36 [62]. Typical participants were males in their 40s, 50s, and 60s. Studies followed participants immediately after treatment [50, 57, 58, 62] to one month [51, 52, 54–56].

Table 3 describes study characteristics related to intervention and comparators and assessed outcomes. Of the 16 included studies, nine trials [14, 50, 52, 54, 55, 59–62] evaluated TBS:

1. Eight trials compared cTBS versus
  - sham cTBS (both groups received conventional rehabilitation training [52, 60]);
  - 1 Hz rTMS, 10 Hz rTMS, and sham rTMS (all groups with addition of routine rehabilitation [55]);
  - sham TBS [14];
  - sham cTBS [54, 59, 61, 62].
2. One trial compared iTBS with 80% resting motor threshold (RMT) versus iTBS 40% RMT [50].

Of the remaining seven studies, four trials [56–58, 64] evaluated tDCS:

1. Two trials compared tDCS over the left (cathodal) and right (anodal) posterior parietal cortex, one versus placebo at an intensity of 2 mA [56] and the other versus sham tDCS [64].
2. One trial [57] compared tDCS dual versus either tDCS single or tDCS sham.
3. One trial [58] compared tDCS versus sham tDCS.

Three further trials [51, 53, 63] evaluated rTMS:

1. One trial [51] compared rTMS with sham rTMS, both plus conventional rehabilitation therapy (neurodevelopmental facilitation techniques).
2. Two trials compared 1 Hz rTMS, one versus 10 Hz rTMS and sham rTMS [53] (both groups received conventional rehabilitation), and the other trial compared 1 Hz rTMS versus sham rTMS [63].

None of the included studies evaluated noninvasive brain stimulations as an adjunct to any type of conventional stroke rehabilitation.

### 3.3. Risk of Bias

**Figure 2:**
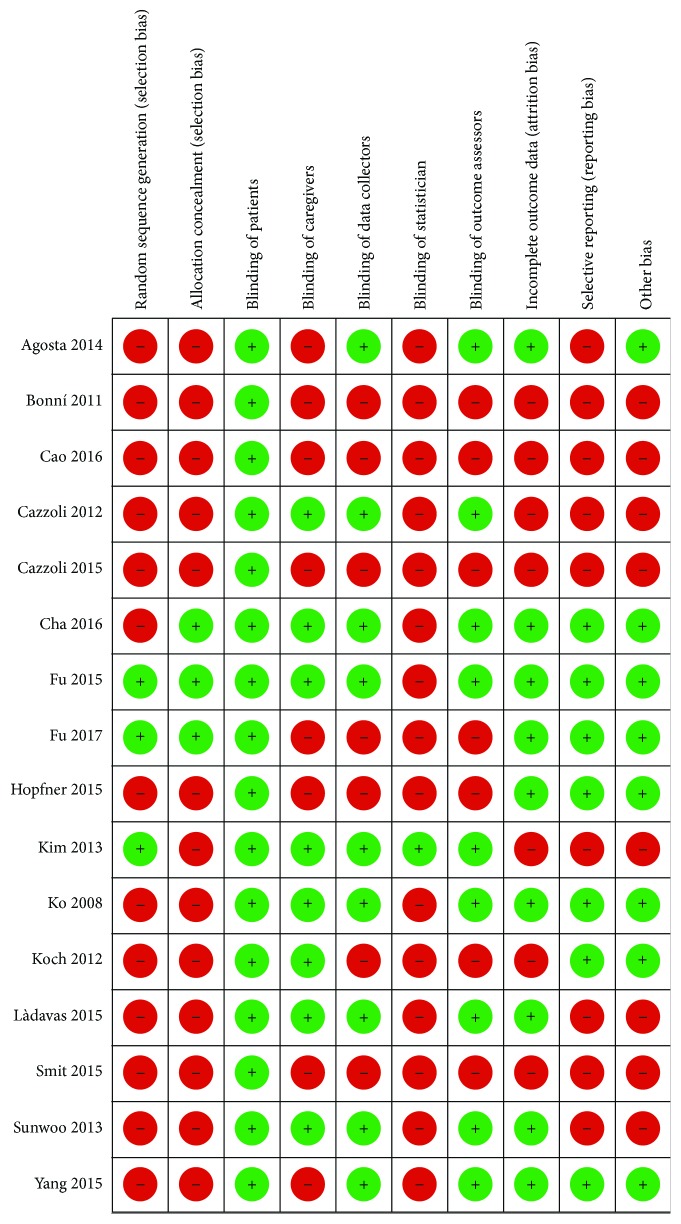
Risk of bias assessment for RCTs and non-RCTs.

Figure 2 describes the risk of bias assessment for the RCTs and non-RCTs, respectively. The major issue regarding risk of bias in the RCTs and non-RCTs was problems of random sequence generation [14, 50, 51, 54–59, 61–64] and concealment of randomization [14, 50, 53–59, 61–64]. An additional problem was blinding of the statistician in all included studies.

### 3.4. Outcomes

#### 3.4.1. Synthesized Results from Randomized Controlled Trials

**Figure 3:**
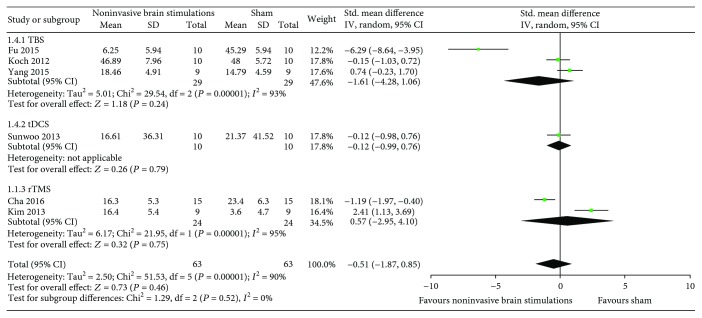
Meta-analysis of overall USN measured by the star cancellation test.

*(1) Overall USN Measured by the Star Cancellation Test*. The results from six RCTs [51–55, 57] comparing noninvasive brain stimulations with sham failed to show a benefit in overall USN measured by the star cancellation test (SMD −0.51, 95% CI −1.87, 0.85; *p* = 0.46; *I* ^2^ = 90%) (Figure 3). The results were consistent regardless of the type of noninvasive brain stimulations (TBS in three RCTs [52, 54, 55] (SMD −1.61, 95% CI −4.28, 1.06; *p* = 0.24; *I* ^2^ = 93%); dual-tDCS in one RCT [57] (SMD −0.12, 95% CI −0.99, 0.76; *p* = 0.79; *I* ^2^ = not applicable); and 1 Hz rTMS in two RCTs [51, 53] (SMD 0.57, 95% CI −2.95, 4.10; *p* = 0.75; *I* ^2^ = 95%)) (Figure 3). Certainty in evidence was rated as very low because of imprecision, inconsistency, and risk of bias due to the studies that were ranked as high risk of bias for both allocation sequence and allocation concealment (Figure 2).

**Figure 4:**
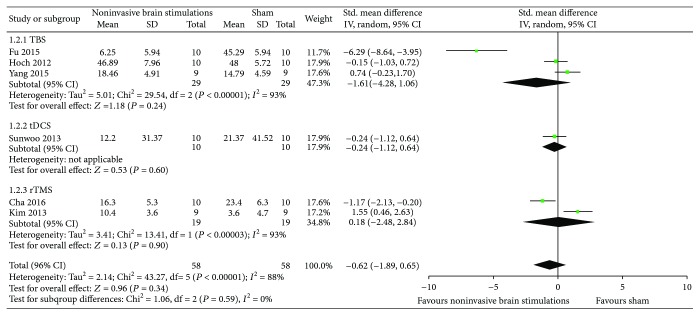
Sensitivity analysis of overall USN measured by the star cancellation test using TBS, single-mode tDCS, and 10 Hz rTMS.

A sensitivity analysis from the same RCTs using TBS [52, 54, 55], single-tDCS [57], and 10 Hz rTMS [51, 53] yielded results that were also consistent with the primary analysis and failed to show a difference in the effects of noninvasive brain stimulations compared to sham (SMD −0.62, 95% CI −1.89, 0.65; *p* = 0.34; *I* ^2^ = 88%) (Figure 4).

**Figure 5:**
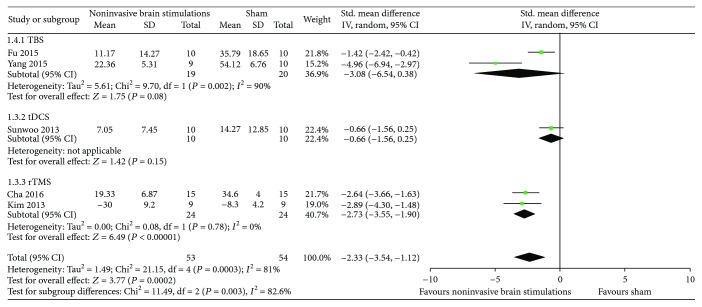
Meta-analysis of overall USN measured by the line bisection test.

*(2) Overall USN Measured by the Line Bisection Test*. Results from five RCTs [51–53, 55, 57] comparing noninvasive brain stimulations with sham suggested a benefit in overall USN measured by the line bisection test (SMD −2.33, 95% CI −3.54, −1.12; *p* = 0.0002; *I* ^2^ = 81%) (Figure 5). The results were inconsistent when the data were analyzed by type of noninvasive brain stimulations: TBS in two RCTs [52, 55] (SMD −3.08, 95% CI −6.54, 0.38; *p* = 0.08; *I* ^2^ = 90%) and dual-tDCS in one RCT [57] (SMD −0.66, 95% CI −1.56, 0.25; *p* = 0.15; *I* ^2^ = not applicable) except by 1 Hz rTMS in two RCTs [51, 53] that yielded results that were consistent with the overall meta-analysis (SMD −2.33, 95% CI −3.54, −1.12; *p* < 0.0002; *I* ^2^ = 81%) (Figure 5). Certainty in evidence was rated as low because of inconsistency and risk of bias due to the studies that were ranked as high risk of bias for both allocation sequence and allocation concealment (Figure 2).

**Figure 6:**
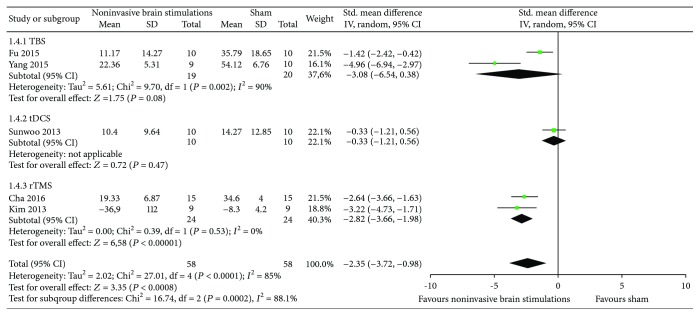
Sensitivity analysis of overall USN measured by the line bisection test using TBS, single-mode tDCS, and 10 Hz rTMS.

A sensitivity analysis from the same RCTs using TBS [52, 55], tDCS [57], and 10 Hz rTMS [53] yielded results that were also consistent with the primary analysis and suggested a difference in the effects of noninvasive brain stimulations compared to sham (SMD −2.35, 95% CI −3.72, −0.98; *p* = 0.002; *I* ^2^ = 85%) (Figure 6).

**Figure 7:**
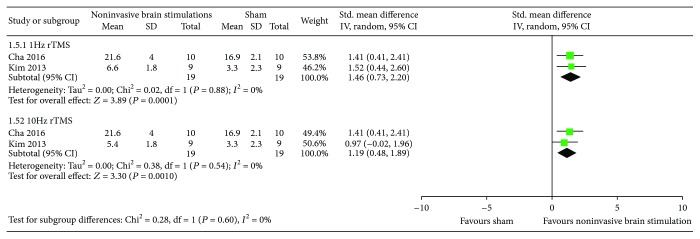
Meta-analysis of overall USN measured by the Motor-Free Visual Perception Test.

*(3) Overall USN Measured by Motor-Free Visual Perception Test*. The results from two RCTs [51, 53] comparing noninvasive brain stimulations with sham suggested a benefit in overall USN measured by the Motor-Free Visual Perception Test at 1 Hz (SMD 1.46, 95% CI 0.73, 2.20; *p* < 0.0001; *I* ^2^ = 0%), and the difference was not observed using 10 Hz (SMD 0.97, 95% CI −0.02, 1.96; *p* = 0.06; *I* ^2^ = not applicable) (Figure 7). Certainty in evidence was rated as moderate because of risk of bias due to the studies that were ranked as high risk of bias for both allocation sequence and allocation concealment (Figure 2).

**Figure 8:**
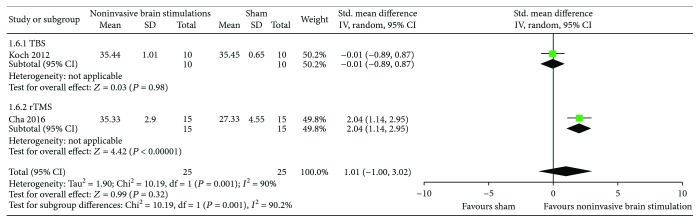
Meta-analysis of overall USN measured by Albert's test and the line crossing test.

*(4) Overall USN Measured by Albert's Test and the Line Crossing Test*. The results from two RCTs [51, 54] comparing noninvasive brain stimulations with sham failed to show a benefit in overall USN measured by Albert's test and the line crossing test (SMD 1.01, 95% CI −1.0, 3.02; *p* = 0.32; *I* ^2^ = 90.2%) (Figure 8). However, in the subgroup analysis with the use of 1 Hz rTMS, we found a statistically significant difference compared to sham (SMD 2.04, 95% CI 1.14, 2.95; *p* < 0.00001; *I* ^2^ = not applicable). Regarding the use of TBS, there was no benefit compared to sham (SMD −0.01, 95% CI −0.89, 0.87; *p* = 0.98; *I* ^2^ = not applicable). Certainty in evidence was rated as low because of inconsistency and risk of bias due to the studies that were ranked as high risk of bias for both allocation sequence and allocation concealment (Figure 2).

*(5) Other Outcomes: Daily Life Functions and Adverse Events*. Only Kim et al. [53] reported on daily life functions with a higher mean in the 10 Hz rTMS group than in the sham and 1 Hz rMTS groups; however, there was only a statistically significant difference favoring the 10 Hz rTMS group compared to the sham group (SMD 1.83, 95% CI 0.68, 2.97; *p* = 0.002; *I* ^2^ = not applicable). Làdavas et al.'s study [64] was the only study that reported on adverse events; no significant adverse effect of tDCS was reported, except only a few cases of minimal irritation of the skin beneath the electrodes.

None of the included studies reported on the following outcomes: neurological and functional disabilities, loss of balance, depression or anxiety and evaluation of poststroke fatigue, quality of life, and death.

#### 3.4.2. Synthesized Results from Non-RCTs

The non-RCTs did not report data in a usable way to allow for any statistical analysis.

## 4. Discussion

### 4.1. Main Findings

Based on pooled data from six randomized trials with 116 participants, we found evidence for a benefit in overall USN with noninvasive brain stimulation, especially with the use of rTMS in comparison to the sham (Figures 5, 7, and 8). The evidence is from moderate-quality evidence because of risk of bias due to the studies that were ranked as high risk of bias for both allocation sequence and allocation concealment (Figure 2). Non-RCT studies provided no evidence, suggesting that future trials should adhere to CONSORT guidelines to ensure clarity and reproducibility in the reporting of methods.

We presented the results of overall USN in a forest plot, which showed a statistically significant difference between the noninvasive brain stimulations and sham in the following tests: line bisection test, Motor-Free Visual Perception Test, and Albert's test and line crossing test. Nevertheless, the study also showed a nonsignificant difference between the noninvasive brain stimulations and sham on the star cancellation test.

Several noninvasive brain stimulations have been explored to determine whether some of these techniques might be useful in promoting recovery from USN after stroke. The lesion of the right parietal cortex after stroke causes disinhibition of the left hemisphere and thus a pathological overactivation of the latter. This overactivation in the left depresses the neural activity by an increased inhibition on the right hemisphere, aggravating the perception. The rTMS can generate currents capable of depolarizing cortical neurons, and tDCS changes cortical activity by means of small electric currents and does not evoke action potentials. The tDCS has the advantage that the device is inexpensive, portable, and easy to use, but rTMS presented more activation of the neural network and induced a neuroplastic response for a long-term potentiation [65].

In three of four meta-analyses, rTMS was responsible for the improvement of overall USN, revealing that an electric current is an effective strategy for generating lasting promising effects in the brain. Unfortunately, we did not find any significant TBS or tDCS effects compared to sham procedures.

### 4.2. Strengths and Limitation

Strengths of our review include a comprehensive search; assessment of eligibility, risk of bias, and data abstraction independently and in duplicate; assessment of risk of bias that included a sensitivity analysis addressing loss to follow-up; and use of the GRADE approach for rating the certainty of evidence for each outcome (Table 3). Furthermore, there were no language restrictions, and translations of non-English trials were obtained whenever possible.

The primary limitation of our review is the low certainty consequent to study limitations. We identified a small number of RCTs with a modest number of participants resulting in wide confidence intervals. The total number of participants was relatively very low (RCTs *n* = 278, non-RCTs *n* = 94) due to the small sample sizes of individual trials, which led to downgrading the quality of evidence in some instances because underpowered trials are likely to have a greater degree of imprecision.

Moreover, selection bias and unblinding were substantial. Another limitation of this review was having an insufficient number of included studies to allow for the complete statistical analysis that we had planned. We were not able to assess publication bias because there were fewer than 10 eligible studies addressing the same outcome in a meta-analysis. We also planned to perform subgroup analyses according to the characteristics of stroke type, type of stimulation, type of frequency, and comparators (type of control intervention, i.e., pharmacological therapy versus nonpharmacological). However, we also were not able to conduct these analyses because they did not meet our minimal criteria, which was at least five studies available with at least two in each subgroup.

Although this review presents several limitations, the issue is whether one should dismiss these results entirely or consider them bearing in mind the limitations. The latter represents our view of the matter.

### 4.3. Relation to Prior Work

The research question we investigated in our review has been addressed before from different perspectives using our population of interest but with a different intervention (i.e., pharmacological intervention) [7] or investigating either the intervention or the control arms explored in this review but with a different population (e.g., idiopathic Parkinson's disease (IPD) [48], panic disorder in adults [66], or amyotrophic lateral sclerosis or motor neuron disease) [13].

Two Cochrane reviews [21, 49] evaluated the effect of tDCS in people after stroke but not in comparison with rTMS; instead, the authors compared tDCS with placebo, sham tDCS, no intervention, or conventional motor rehabilitation. The first review's [49] authors found evidence of effect regarding activities of daily living performance at the end of the intervention period and at the end of follow-up. However, the results did not persist in a sensitivity analysis including only trials of good methodological quality. In the second review [21], the authors found that there were no studies examining the effect of tDCS on cognition in stroke patients with aphasia.

Another Cochrane review [22] that addressed the use of rTMS compared to sham treatment or other conventional treatment for improving function after stroke revealed that rTMS treatment was not associated with improved activities of daily living, nor did it have a statistically significant effect on motor function.

Three additional Cochrane reviews also discussed the effects of both tDCS [48] and rTMS [13, 66] but in different populations—in Parkinsonism [48], in patients with amyotrophic lateral sclerosis or motor neuron disease [13], and in adults with panic disorder [66]. All reviews suffered from poor methodological quality, imprecision, and hence low confidence in the estimate of the true effect to draw a consistent conclusion on the effects of noninvasive brain stimulations.

### 4.4. Implications

Moderate-quality evidence shows that rTMS, at 1 Hz, is more efficacious than sham for unilateral spatial neglect after stroke measured by Motor-Free Visual Perception Test. Furthermore, low-quality evidence also suggests a benefit of noninvasive brain stimulation, particularly with the use of rTMS, for overall USN measured by the line bisection test, Albert's test, and the line crossing test. Future trials should adhere to CONSORT guidelines to ensure clarity and reproducibility in the reporting of methods [67].

## Figures and Tables

**Table 1:** GRADE evidence profile for RCTs: noninvasive brain stimulations for unilateral spatial neglect after stroke.

Quality assessment						Illustrative comparative risks (95% CI)		Certainty in estimates orquality of evidence
						Assumed risk	Corresponding risk	
Number of participants (studies) Range follow-up Time in weeks	Risk of bias	Inconsistency	Indirectness	Imprecision	Publication bias	Sham	Noninvasive brain stimulations	
Overall USN measured by star cancellation test								
116 (6) Immediately postintervention 4 weeks	Serious limitation^1^	Serious limitation^2^	No serious limitation^3^	Serious limitation^4^	Undetectable	The mean in change in USN measured by star cancellation test was 45.29 (SD 5.94)^∗^	The std. mean in changes in USN measured by star cancellation test in the intervention group was on average 0.51 fewer (1.89 fewer to 0.88 more)	Very low

Overall USN measured by line bisection test								
107 (5) Immediately postintervention 1 month	Serious limitation^1^	Serious limitation^2^	No serious limitation^3^	No serious limitation	Undetectable	The mean in change in USN measured by line bisection test was 35.79 (SD 18.65)^∗^	The std. mean in changes in USN measured by line bisection test in the intervention group was on average 2.33 fewer (3.54 fewer to 1.12 fewer)	Low

Overall USN measured by Motor-Free Visual Perception Test								
38 (2) 2–4 weeks	Serious limitation^1^	No serious limitation	No serious limitation	No serious limitation	Undetectable	The mean in change in USN measured by Motor-Free Visual Perception Test was 16.9 (SD 2.1)^∗∗^	The std. mean in changes in USN measured by Motor-Free Visual Perception Test in the intervention group was on average 1.46 more (0.73 more to 2.20 more)	Moderate

Overall USN measured by Albert test and line crossing test								
50 (2) 4 weeks	Serious limitation^1^	Serious limitation^2^	No serious limitation	No serious limitation	Undetectable	The mean in change in USN measured by Albert test and line crossing test was 27.33 (SD 4.55)^∗∗^	The std. mean in changes in USN measured by Albert test and line crossing test in the intervention group was on average 1.01 more (1 fewer to 3.02 more)	Low

SD = standard error; std. = standardized. ^∗^Baseline risk estimates for overall USN come from control arm of Fu et al.'s [52] study (lowest risk of bias trial in the meta-analysis). ^∗∗^Baseline risk estimates for overall USN come from control arm of Cha et al.'s [51] study (newest trial in the meta-analysis). ^1^The majority of the studies were ranked as high risk of bias for both allocation sequence and allocation concealment. ^2^There was a substantial heterogeneity (*I* ^2^> 70%). ^3^There was no substantial difference related to the mean age and eligibility criteria throughout the six included studies. ^4^95% CI for absolute effects includes clinically important benefit and no benefit.

**Table 2:** Study characteristics related to design of study, setting, number of participants, mean age, gender, and inclusion and exclusion criteria.

Author, year	Design of study	Status of publication	Location	No.^∗^ of participants	Mean age	No. of males (%)	Inclusion criteria	Exclusion criteria
Randomized controlled trials								
Cao et al., 2016 [50]	Parallel RCT	Full text	Asia	I: 7 C: 6	I: 55.0 C: 62.0	I: 85.7 C: 83.3	Right-handed patients who had a first-ever stroke in the right hemisphere and visuospatial neglect with normal or corrected-to-normal vision	NR
Cha and Kim, 2016 [51]	Parallel RCT	Full text	Asia	I: 15 C: 15	I: 64.07 C: 63.33	I: 64 C: 60.0	Had a first right hemisphere stroke (cerebral infarction or hemorrhage) more than 2 weeks before the study, which had been confirmed by computed tomography or magnetic resonance imaging (MRI); had VSN determined by line bisection tests (rightward bias > 12%) or star cancelation test (omission of any number of stars); had a Glasgow coma scale score < 15; 18–80 years old; right-handed; normal vision or normal corrected vision; and had the ability to understand the study and signed an informed consent form	All patients did not have brain tumors or other brain pathology. Excluded were patients with hemianopia; subarachnoid hemorrhage, venous sinus thrombosis, transient ischemic attack, reversible ischemia, or a condition exacerbated by a new infarction or hemorrhage site; a medical history or family history of seizure; or with metal devices or claustrophobia preventing MRI
Fu et al., 2015 [52]	Parallel RCT	Full text	Asia	I: 11 C: 11	I: 55.1*^β^* C: 59.5*^β^*	I: 80.0 C: 80.0	Right-handed patients with right hemisphere stroke (hemorrhagic or ischemic lesion) confirmed by computed tomography or magnetic resonance imaging > 2 weeks before the beginning of the study and diagnosis of visuospatial neglect based on clinician judgement and on deficits in at least one out of two paper-pencil tests	Age < 30 years or > 80 years, history of epilepsy, previous head trauma, drug and alcohol abuse and psychiatric disorders, recurrent stroke, obvious aphasia and communication obstacles, family history of seizures, ever use of tricyclic antidepressants or antipsychotic drugs, diamagnetic metal implants such as cardiac pacemakers, and visual field defects
Fu et al., 2017 [60]	Parallel RCT	Full text	Asia	I: 6 C: 6	I: 60.17 C: 62	I: 75 C: 75	Had a first right hemisphere stroke (cerebral infarction or hemorrhage) more than 2 weeks before the study, which had been confirmed by computed tomography or magnetic resonance imaging (MRI); had VSN determined by line bisection tests (rightward bias > 12%) or star cancelation test (omission of any number of stars); had a Glasgow coma scale score < 15; 18–80 years old; right-handed; normal vision or normal corrected vision; and had the ability to understand the study and sign an informed consent form; all patients did not have brain tumors or other brain pathology	Patients with hemianopia; subarachnoid hemorrhage, venous sinus thrombosis, transient ischemic attack, reversible ischemia, or a condition exacerbated by a new infarction or hemorrhage site; a medical history or family history of seizure; or with metal devices or claustrophobia preventing MRI
Smit et al., 2015 [56]	RCT cross-over study	Full text	Europe	I: 5^€^ C: 5^€^	I: 64.8^€^ C: 64.8^€^	I: 60.0^€^ C: 60.0^€^	Patients with left hemispatial neglect after right-hemispheric lesion, right-handed, older than the age of 18, more than four months after stroke	Patients with severe language and communication disorders, bilateral cortical damage, psychiatric disorders, alcohol and/or drug addiction, epilepsy, eczema or damages on the scalp, metal or other foreign parts in the head
Yang et al., 2015 [55]	Parallel RCT	Full text	Asia	I: 9 I2: 10 I3: 9 C: 10	I: 46.7 I2: 48.0 I3: 49.4 C: 47.7	I: 66.6 I2: 40.0 I3: 55.6 C: 30.0	Age between 18 and 80; first stroke patients (cerebral infarction or hemorrhage) and in recovery time within 60–180 days; USN confirmed by line bisection test, star cancellation test, or clinical examination; no metallic implant of diamagnetic substance; signed the informed consent	Subarachnoid hemorrhage, venous sinus thrombosis, and reversible or transient ischemic attacks; worsening condition and new-onset infarction or hemorrhage; GCS score < 15; obvious aphasia and severe cognitive-communicationdisorders; family history of epilepsy; impaired organ function or failure in the heart, lung, liver, kidney, or other vital organs and life expectancy < 6 months; history of claustrophobia and uncooperative during examination; and hemianopsia
Kim et al. [53]	Parallel RCT	Full text	Asia	I: 9 I2: 9 C: 9	I: 68.6 I2: 64.1 C: 68.3	I: 55.6 I2: 44.4 C: 66.7	Patients with right cerebral ischemic or hemorrhagic with visuospatial neglect (confirmed using the line bisection test); all patients were right-handed	Severe cognitive impairment making them unable to understand the instructions; contraindications for TMS, such as a history of epileptic seizure, major head trauma, and presence of metal in the skull or pacemaker; or unstable medical or neurologic conditions
Sunwoo et al., 2013 [57]	RCT cross-over study	Full text	Asia	I: 10 I2: 10 C: 10	62.6^¢^	40.0*^β^*	Stroke patients with lesion in the right hemisphere involving the parietal cortex, and left USN diagnosed by clinical observation and confirmed by a line bisection test; all patients were previously right-handed	Patients who had metallic implants in the cranial cavity, a skull defect, history of seizure, uncontrolled medical problems, and severe cognitive impairment
Cazzoli et al., 2012 [14]	Parallel RCT	Full text	Europe	24^£^	58.0^¢^	70.8^¢^	Ischemic or hemorrhagic lesion to the right hemisphere and left-sided spatial neglect determined on the basis of deficits in at least two out of three classes of paper-pencil tests and on clinical judgement; all patients had to have normal or corrected-to-normal visual acuity	History of epilepsy, prior head trauma, drug and alcohol abuse, and major psychiatric disorders
Ko et al., 2008 [58]	RCT cross-over study	Full text	Asia	I: 15^€^ C: 15^€^	I: 62.1^€^ C: 62.1^€^	I: 66.6^€^ C: 66.6^€^	Patients with subacute stroke with neglect	Patients who had metal in the cranial cavity or calvarium, skin lesions in the area of electrode, uncontrolled medical conditions, and severe cognitive impairments
Koch et al., 2012 [54]	Parallel RCT	Full text	Europe	I: 10 C: 10	I: 61.4^#^ C: 71.9^#^	I: 55.5^#^ C: 55.5^#^	Right-handed patients, with right hemisphere subacute ischemic stroke affected by hemispatial neglect, confirmed by radiologic (CT or MRI) and clinical examination	NR
Bonnì et al., 2011 [59]	Parallel RCT	Conference abstract	Europe	NR	NR	NR	Subacute stroke patients with neglect	NR
Non-RCTs								
Cazzoli et al., 2015 [61]	Non-RCT cross-over study^§^	Full text	Europe	I: 8^¥^ C: 8^¥^	I: 52.6 and 54.2*^α^* C: 53.0	NR	Patients with left-sided, hemispatial neglect after a subacute right-hemispheric stroke; all patients had normal or corrected-to-normal visual acuity	Not clearly reported, however, authors have assessed patients by means of internationally accepted safety guidelines for the application of TMS, which included screening for a history of epilepsy, prior head trauma, drug and alcohol abuse, and major psychiatric disorders
Hopfner et al., 2015 [62]	Non-RCT cross-over	Full text	Europe	I: 18^€^ C: 18^€^	I: 64.5^€^ C: 64.5^€^	I: 50.0^€^ C: 50.0^€^	Left-sided neglect, based on clinical judgement and neuropsychological testing, after subacute right-hemispheric stroke; all subjects had normal or corrected-to-normal visual acuity	NR
Làdavas et al., 2015 [64]	Quasi-RCT	Full text	Europe	I: 8 I2: 11 C: 11	I: 72.0 I2: 66.0 C: 67.0	I: 50.0 I2: 54.5 C: 54.5	Patients with right hemisphere stroke with hemispatial neglect and performance on the Behavioral Inattention Test battery with scores ≤ 129	Presence of widespread mental deterioration (Mini-Mental State Examination score < 20), psychiatric disorders, a history of prior stroke or hemorrhage, any severe internal medical disease, epilepsy, and additional factors influencing the risk of epilepsy
Agosta et al., 2014 [63]	Non-RCT cross-over study	Full text	Europe	I: 6^€^ C: 6^€^	I: 67.83^€^ C: 67.83^€^	I: 66.6^€^ C: 66.6^€^	Patients with right hemisphere unilateral lesions due to a cerebrovascular stroke, confirmed by radiological examination (CT or MR), in their chronic stage after the stroke (at least six months post onset); besides, participants were right-handed, native Italian speakers, and had normal or corrected-to-normal visual acuity	History or evidence of degenerative disease or psychiatric disorder

C: control group; CT: computed tomography; GCS: Glasgow coma scale; I: intervention; MR: magnetic resonance imaging; No.: number; RCT: randomized controlled trial; TMS: transcranial magnetic stimulation; USN: unilateral spatial neglect. ^€^Participants of the experimental group also served as controls. ^¥^Five patients were randomized in parallel design, and three further patients included in both groups. ^£^The authors did not specify the sample size per studied group. *^α^*Data comprises three patients that received both experimental and control interventions. *^β^*Data was calculated from 10 patients (one patient was excluded after randomization). ^¢^Data are from the whole sample, as the authors did not specify it per studied group. ^#^Data are from 9 patients in each group. ^§^The study was a cross-over for only three patients, for the remaining ten patients the study was a RCT.

**Table 3:** Study characteristics related to intervention and control groups, assessed outcomes, and follow-up.

Author, year	Description of interventions	Description of control groups	Measured outcomes	Follow-up
Randomized controlled trials				
Cao et al., 2016 [50]	**iTBS** 80% RMT in the rTMS group: stimulation was applied using an 87 mm butterfly coil connected to a Magstim Rapid2 (Magstim Co., Whitland, UK), with peak intensity of 2.0 T and a maximum pulse length of 250 *μ*s. Pulses (theta burst type) were delivered to the left dorsal lateral prefrontal cortex, the F5 label of the left hemisphere, which is between the F3 and F7, at 80% of resting motor threshold. Two sessions were applied with a 15 min interval on each day. Each session included 20 stimulation trains consisting of three pulses delivered at a frequency of 50 Hz in every 200 ms for 2 s (total 10 bursts, 30 pulses) with an interval of 8 s.	Same as intervention group; however, pulses were delivered at 40% of RMT	Line bisection and star cancellation tests	After intervention
Cha and Kim, 2016 [51]	**Repetitive rTMS** + conventional rehabilitation therapy (neurodevelopmental facilitation techniques) for a total of 40 minutes (rTMS: 10 min; rehabilitation: 30 min) per day, with a 10-minute rest period halfway through the session, for 4 weeks, 5 days per week: stimulation was delivered using figure-of-eight coil with a diameter of 80 mm connected to Magstim Rapid2 (Magstim Co. Ltd., Wales, UK). Stimulation was applied in the right posterior parietal (P3 and P4 areas) based on the electroencephalogram 10/20 system at a frequency of 1 Hz for 5 minutes with 90% of the motor threshold during rest.	Sham rTMS and conventional rehabilitation therapy using the same protocol than the experimental group	Motor-Free Visual Perception Test; line bisection test; Albert test; star cancellation test	4 weeks
Fu et al., 2015 [52]	Left posterior parietal cortex **cTBS** + conventional rehabilitation training: cTBS was set over P5, three-pulse burst was delivered at 30 Hz and repeated every 200 ms for 40 s with intensity was 80% of the resting motor threshold. cTBS was delivered using a Super Rapid 2 magnetic stimulator (Magstim, Whitland, UK) with 2.0-Tesla maximum field strength, connected with a figure-of-eight coil (diameter of outside loop, 87 mm). Patients received 4 trains daily, with an interval of 15 min, for 14 consecutive days.	Sham cTBS + conventional rehabilitation training	Star cancellation test; line bisection test	4 weeks
Fu et al., 2017 [60]	The cTBS group received continuous TBS with the coil placed tangentially to the scalp at P3 over the left posterior parietal cortex (according to the 10–20 electrode position system of the American Electroencephalographic Association28). The magnitude of the pulses was maintained at 80% resting motor threshold. On each day for 10 consecutive days, 4 sessions of stimulation were delivered, with an interval of 15 min between every 2 sessions. Each session lasted 40 s and contained 600 pulses delivered in 200 bursts at 5 Hz (theta rhythm). Each burst included 3 pulses delivered at 30 Hz.	The active control group received stimulations with the same features at the same position as the cTBS group, but with the coil placed perpendicular to the scalp surface and the amplitude of the stimulation pulses reduced to 40% resting motor threshold	Star cancellation test; line bisection test	10 days
Smit et al., 2015 [56]	**tDCS** was applied for 20 minutes over the left (cathodal) and right (anodal) posterior parietal cortex on five consecutive days with a battery-driven direct current stimulator (NeuroConnDC-Stimulator; serialnumber 0096). Stimulation parameters were set at a current of 2000 mA, and a resistance of <10 kOhm, applied for 1200s with ramping up in 30 s and ramping down in 30 s. Electrodes were located over the posterior parietal lobe, corresponding with P3 (cathodal electrode) and P4 (anodal electrode). Treatment conditions were separated by a four-week washout period.	Placebo was applied for 20 minutes over the left (cathodal) and right (anodal) posterior parietal cortex at an intensity of 2 mA on five consecutive days; treatment conditions were separated by a four-week washout period	Cancellation tests; line bisection tests; drawing tests	1 month
Yang et al., 2015 [55]	Group I: 1 Hz **rTMS** two times a day for 2 weeks + routine rehabilitation: stimulation was administered using a rapid magnetic stimulator (Magstim Company) with a figure-of-eight coil, peak intensity of stimulation at 2 T, and pulse duration of 250 s, at the contralateral hemisphere (P3), intensity 80% of RMT, and frequency of 1 Hz, and stimulus duration for each sequence was 8 s, repeated 82 sequences with a total of 656 pulse numbers. Group I2: 10 Hz **rTMS** two times a day for 2 weeks + routine rehabilitation: stimulation was administered using a rapid magnetic stimulator (Magstim Company) with a figure-of-eight coil, peak intensity of stimulation at 2 T, and pulse duration of 250 s, at the contralateral hemisphere (P3), intensity 80% of RMT, frequency 10 Hz, with a total pulse number of 1000 and stimulation interval of 55 s. Group I3: **cTBS** two times a day for 2 weeks + routine rehabilitation: stimulation was administered using a rapid magnetic stimulator (Magstim Company) with a figure-of-eight coil, peak intensity of stimulation at 2 T, and pulse duration of 250 s, at the contralateral hemisphere (P3), intensity 80% of RMT, 801 pulses, in bursts of 3 pulses at 30 Hz, repeated every 100 ms.	Sham rTMS two times a day for 2 weeks + routine rehabilitation	Star cancellation test; line bisection test	1 month
Kim et al., 2013 [53]	Group A: 10 sessions of low-frequency (**1 Hz**) **rTMS** over the nonlesioned left posterior parietal cortex (P3) at a 90% motor threshold in 4 trains of 5-minute duration, each separated by 1 minute (resulting in a total stimulation period of 20 minutes). rTMS was delivered using a Magstim Super Rapid Magnetic Stimulator with a 70-millimeter, air-cooled 8-shaped coil. rTMS was performed 5 times per week for 2 weeks. Patients also received conventional rehabilitation treatment (including physical, occupational, and cognitive therapies). Group B: 10 sessions of high-frequency (**10 Hz**) **rTMS** over the lesioned right posterior parietal cortex (P4) at a 90% motor threshold in 4 trains of 5-minute duration, each separated by 55 seconds (resulting in a total stimulation period of 20 minutes). The remaining of the protocol followed the same instructions as group A.	Sham rTMS + conventional rehabilitation	Motor-Free Visual Perception Test; line bisection test; cancellation test; Catherine Bergego scale; Korean-modifiedBarthel index	2 weeks
Sunwoo et al., 2013 [57]	Group A: dual-mode (**tDCS dual**) direct current was delivered by two sets of battery-powered devices (Phoresor II Auto Mod-elPM850, IOMED, USA) using two pairs of surface saline-soaked sponge electrodes (5 cm × 5 cm). Anodal tDCS of the first circuit over the right PPC (P4) was accompanied by cathodal tDCS of the second circuit over the left PPC (P3). Therefore, in the first tDCS circuit, the anode was placed over P4 and the cathode was placed over the left supraorbital area. In the second tDCS circuit, the anode was placed over the right supraorbital area and the cathode was placed over the P3. A constant current of 1 mA was delivered for 20 min. Group B: Single-mode (**tDCS single**) direct current was delivered by two sets of battery-powered devices (Phoresor II Auto Mod-elPM850, IOMED, USA) using two pairs of surface saline-soaked sponge electrodes (5 cm × 5 cm). The anode was placed over P4 and the cathode over the left supraorbital area (the first tDCS circuit), and real stimulation was provided, whereas the second tDCS circuit received sham stimulation. For the real stimulation, a constant current of 1 mA was delivered for 20 min. For the sham stimulation, the stimulator was turned on and the current intensity was gradually increased for 5 s, and was then tapered off over 5 s.	Sham mode (tDCS sham) in the first and second tDCS circuits. The stimulator was turned on and the current intensity was gradually increased for 5 s, and was then tapered off over 5 s	Line bisection test; star cancelation test	Immediately after treatment
Cazzoli et al., 2012 [14]	**cTBS** for 2 days on week 1 and sham TBS for 2 days on week 2. cTBS was applied by means of a MagPro X100 stimulator (Medtronic Functional Diagnostics) connected to a round coil with 60 mm outer radius (Magnetic Coil Transducer MC-125). cTBS protocol comprised 801 pulses, delivered in a continuous train and consisting of 267 bursts, each one contained three pulses at 30 Hz, repeated at 6 Hz (total duration of one single, cTBS train was 44 s), and eight cTBS trains were applied over 2 days. cTBS was applied over P3. Besides, patients received neurorehabilitation therapy including 1 h neuropsychological training, 1 h of occupational therapy, and 1 h of physiotherapy per day.	Control A: sham TBS for 2 days on week 1 and cTBS for 2 days on week 2. cTBS protocol was the same described for intervention A. Besides, patients received neurorehabilitation therapy including 1 h neuropsychological training, 1 h of occupational therapy, and 1 h of physiotherapy per day Control B: sham TBS for 2 days on week 1 and sham TBS for 2 days on week 2. Besides, patients received neurorehabilitation therapy including 1 h neuropsychological training, 1 h of occupational therapy, and 1 h of physiotherapy per day	Catherine Bergego scale; Vienna Test System; random shape cancelation test	2 weeks
Ko et al., 2008 [58]	**tDCS** to the right posterior parietal cortex for 20 min (2 mA anodal DC brain polarization) delivery by a battery-powered device (Phoresor II Auto model PM850, IOMED, USA), using a pair of saline-soaked surface sponge electrodes (5 cm × 5 cm). The anode was placed over P4, and cathode was placed over left supraorbital area.	Sham tDCS (current was delivered for 10 s and then turned off)	Line bisection test; shape-unstructuredcancellation test; letter-structuredcancellation test	Immediately post intervention
Koch et al., 2012 [54]	**cTBS** was delivered using a MagStim Super Rapid magnetic stimulator (Magstim Company, Whitland, Wales, UK), connected with a figure-of-eight coil with a diameter of 70 mm. In each session, 3-pulse bursts at 50 Hz repeated every 200 ms for 40 s were delivered at 80% of the active motor threshold over the left PPC (600 pulses). Every day, 2 sessions of left PPC cTBS were applied with an interval of 15 minutes and lasted for 10 days (5 days per week, Monday to Friday). Patients also received rehabilitation program consisted of 20 sessions of 45 minutes each, held 5 days per week (based on computerized visuospatial scanning training) and motor rehabilitation when necessary.	Sham cTBS was delivered with the coil angled at 90°, with only the edge of the coil resting on the scalp Stimulus intensity, expressed as a percentage of the maximum stimulator output, was set at 80% of the active motor threshold inducing the same acoustic sensation as for real TBS Patients also received rehabilitation program	Line crossing test; letter cancellation test; star cancellation test; figure and shape copying test; representative drawing test	1 month
Bonnì et al., 2011 [59]	**cTBS** over the left PPC, for two weeks.	Sham cTBS	Standardized behavioural inattention test; excitability of the parieto-frontalfunctional connections	NR

Non-RCTs				
Cazzoli et al., 2015 [61]	**cTBS** over the left, contralesional PPC (P3), was applied using a MagPro X100 stimulator, connected to either a round coil (MC-125 Medtronic Functional Diagnostics). The cTBS protocol consisted of 801 pulses delivered in a continuous train. The train was comprised of 267 bursts, where each contained three single pulses at 30 Hz, repeated at 6 Hz, and had a total duration of 44 s. Application consisted on two trains separated by a 15 min interval.	Sham cTBS over the left, contralesional PPC, was applied using a sham coil (MC-P-B70 Medtronic Functional Diagnostics)	Computerised balloon test with eye movement recording; paper-pencil cancellation tasks	8 hours
Hopfner et al., 2015 [62]	**cTBS** comprised 801 pulses, delivered in a continuous train of 267 bursts (each including 3 pulses at 30 Hz, repeated at 6 Hz). The total duration of a single cTBS train was 44 s. Two cTBS trains were applied overP3, with an intertrain interval of 15 min. A MagPro X100 stimulator (Medtronic Functional Diagnostics, Farum, Denmark), connected to around coil (Magnetic Coil Transducer MC-125) was used to deliver biphasic, repetitive magnetic pulses. Besides, 12 (from 18) patients also received smooth pursuit eye movement training.	Sham cTBS connected to a placebo coil (Magnetic Coil Transducer MC-P-B70) Besides, 12 (from 18) patients also received Smooth pursuit eye movement training	Center of cancellation score; x-position of leftmost cancelled target; number of cancelled targets	Right after treatment
Làdavas et al., 2015 [64]	Group A: 2-week rehabilitation program consisted of 10 sessions of **cathodal tDCS** lasting 30 minutes each and held 5 days per week. tDCS was applied using a battery-driven Eldith (neuroConn GmbH, Ilmenau, Germany) Programmable Direct Current Stimulator with a pair of surface saline-soaked sponge electrodes. In each session, a constant current of 2 mA intensity (current density: 0.57 mA/cm2) was delivered lasting 20 minutes of cathodal tDCS of the left, intact PPC (over P5). Group B: 2-week rehabilitation program consisted of 10 sessions of **anodal tDCS** lasting 30 minutes each and held 5 days per week. The anodal tDCS was placed over the PPC of the damaged hemisphere (P6). The remaining protocol was the same used in group A.	Sham tDCS (montage used in the sham group mimicked that used in the two active groups)	Behavioral Inattention Test	Final follow-up within the first week after the last session
Agosta et al., 2014 [63]	A 10-minute train of repetitive low-frequency (**1** Hz) **rTMS** over the left parietal lobe (P3 site) identified using the 10/20 EEG measurement system. The stimulus was delivered using a 70 mm figure-of-eight coil connected to a Magstim Rapid2 (Magstim Co., UK). Stimulation strength was set to 90% of the threshold to evoke motor responses at rest.	Sham rTMS over the intact left parietal cortex	Visual tracking task; unilateral and bilateral tasks	30 minutes

C: control group; cTBS: continuous theta burst stimulation; I: intervention; iTBS: intermittent theta burst; PPC: posterior parietal cortex; RMT: resting motor threshold; rTMS: repetitive transcranial magnetic stimulation; tDCS: transcranial direct current stimulation; TBS: theta burst stimulation; USN: unilateral spatial neglect.
